# Design, Optimization, and Realization of a Magnetic Multi-Layer Quasi-Zero-Stiffness Isolation Platform Supporting Different Loads

**DOI:** 10.3390/ma18071676

**Published:** 2025-04-06

**Authors:** Shuaijie Yang, Xiuting Sun, Jiawei Qian, Jian Xu, Kaixiang Li

**Affiliations:** 1 School of Aerospace Engineering and Applied Mechanics, Tongji University, Shanghai 200092, China; yangshuaijiewasd@tongji.edu.cn (S.Y.); qianjiawei@tongji.edu.cn (J.Q.);; 2 Aircraft Strength Research Institute of China, Xi’an 710065, China

**Keywords:** Multi-layer Quasi-Zero-Stiffness property, variable load isolation platform, magnetic nonlinearity, bi-objective Pareto optimization

## Abstract

This study presents a Multi-layer Quasi-Zero-Stiffness (ML-QZS) vibration isolation platform for variable loads in large-amplitude and low-frequency dynamic environments. In one isolation mount of the proposed ML-QZS isolation platform, Multi-layer permanent magnets are constructed to generate discontinuous Multi-layer negative-stiffness regions. The first design criterion is to achieve the low-frequency and wide-amplitude vibration isolation range for different loads. The second design criterion is carried out for the dynamic performances of transient and steady states. Since both structural design and damping determine vibration transient time and the displacement transmissibility, which often exhibit contradictions depending on system parameters, a bi-objective Pareto optimization criterion is proposed to balance the vibration transients between different layers while ensuring significant isolation effectiveness in one layer. Finally, the relevant experimental prototype is constructed, and the results verify the design principle of Multi-layer double magnetic ring construction and optimization criterions for structural parameters and damping coefficients. This study provides an advanced nonlinear isolation platform with a wide QZS range for different loads, and the optimization method to coordinate the vibration performances, which provides important theoretical and experimental guidance for the design and realization of isolation platforms in practical engineering applications for large-amplitude and low-frequency dynamic environments.

## 1. Introduction

Vibration is a common phenomenon in engineering applications detrimental to the accuracy and service life of equipment [1,2]. To mitigate vibrations from the environment, vibration isolators are usually installed between the excitation source and protective equipment to suppress environmental vibrations [3,4,5]. In previous studies, linear vibration isolators usually consist of a linear spring and a viscous damper. It is known that linear vibration isolators can suppress excitations with frequencies greater than $\sqrt{2}$ times the natural frequency [6,7]. As the natural frequency of the linear vibration isolator is reduced by increasing the loading mass or decreasing the spring stiffness, an increase in mass or reduction in stiffness may result in large initial installation deformation and even lead to instability. To resolve this problem, various design principles of the Quasi-Zero-Stiffness (QZS) isolators are proposed [8,9,10]. In recent years, various types of QZS isolators have been applied in different dynamic environments. Typical QZS isolators are commonly composed of a positive-stiffness component and a negative-stiffness component. The negative-stiffness structure mainly includes triple springs [11,12,13], cam-rollers [14,15,16], permanent magnets and electromagnets [17,18,19], buckled-beams [20,21,22], bionic structures [23,24,25], X-shaped structures [26,27,28], and other structures [29,30]. The positive-stiffness structure mainly includes springs [31], permanent magnets [32], flexible rods or beams [33,34], and others [35]. The connection between any positive-stiffness mechanism and any negative-stiffness mechanism in parallel can generate the QZS property. In QZS isolators, the positive elastic components are usually assembled in the direction of gravity to bear the load, and then, other elastic components are assembled at the equilibrium. Because the negative-stiffness components always result in nonlinearity, typical QZS isolators realize high loading capacity and low dynamic stiffness at equilibrium, known as the High-Static-Low-Dynamic-Stiffness (HSLDS) characteristic. Unfortunately, QZS isolators have the QZS property at the equilibrium point but exhibit a narrow QZS range around it. Since the nonlinearity cannot be ignored, the vibration characteristics depend on both structural parameters and vibration states, and the multi-steady states phenomenon occurs with increasing excitation amplitude. Also, since the system has very low dynamic stiffness around the equilibrium, a variable load can easily break the designed QZS property and increase the initial frequency required for effective isolation. Therefore, it meets two challenging requirements in one isolation system: extending the amplitude range for the QZS property and improving adaptation to increasing loads.

To address the first requirement—expanding the QZS range to isolate large-amplitude, low-frequency vibration excitations—various design methods and bionic structures are proposed. The first design method to increase the QZS range is reducing the nonlinear characteristics of negative stiffness to compensate for linear positive stiffness. Kovacic et al. [36] proposed a triple-spring mechanism consisting of two nonlinear pre-stressed oblique springs and a linear spring. Compared to typical QZS isolators consisting of one pair of pre-deformed springs, two nonlinear pre-deformed oblique springs can provide a larger range for linear negative stiffness. And, by using the cam-roller mechanism instead of elastic components, the nonlinear strength can be reduced to achieve a wide-range QZS property [37,38,39]. The second design method to increase the QZS range is to employ nonlinear positive stiffness to match the nonlinear negative stiffness. Yu et al. [40] proposed a novel nonlinear stiffness-modulated anti-vibration structure consisting of a disc spring group and a volute spring. The nonlinear positive stiffness provided by the volute spring compensates for the negative stiffness provided by the disc spring group to construct QZS characteristics. Their nonlinearities can weaken each other, which can lead to a wide-range QZS property. Yan [41] et al. utilized a pair of magnets to generate hardening positive stiffness to compensate for the negative stiffness generated by the diamond structure. The third design method to increase the QZS range is to reduce both linear and nonlinear stiffness coefficients close to zero, and the typical novel mechanism is the bio-inspired structures [42,43,44]. Unfortunately, for the three design methods above, the QZS property is still achieved around the equilibrium. Recently, a novel method was proposed, which utilizes multiple bistable negative-stiffness structures connected in parallel with positive-stiffness structures to form a multi-point QZS property. Compared to single-point zero-stiffness design, the double-point zero-stiffness design has a wider range for the QZS property. Gatti et al. [45] and Zhao et al. [46] added a pair of oblique springs to the triple-springs isolation system, which results in a stiffness curve with double zero-stiffness points. Around the double zero-stiffness points, there occurs a region with small stiffness, defined as the wide range for the QZS property. Further, by adding more pairs of pre-stressed oblique springs into the triple-spring mechanism, the stiffness curve of the QZS isolator has more zero-stiffness points [47,48]. The zero-stiffness points can be connected to form a wide-range QZS region through reasonable configuration.

For the second requirement of sufficient loading capacity, the typical QZS design usually fails under variable loads. Chen et al. [49] discovered that even a slight load variation could induce a significant impact on the vibration isolation performance of the QZS isolation system through a triple-spring mechanism, since a slight variation in load could result in a deviation of equilibrium from the original position. However, in engineering practice, loads typically vary over a wide range. Xu et al. [50] designed a magnetic isolator that can adjust the load mass within a certain range by adjusting the initial length of the pre-deformed springs. Chen et al. [51] proposed a low-frequency vibration isolator consisting of disk positive-stiffness spring and a pair of buckling Euler beams with adjustable buckling strength that provide adjustable negative stiffness. Furthermore, Jiao et al. [52] proposed a QZS isolator consisting of a cam-roller negative-stiffness system and a positive-stiffness permanent magnetic spring. The load can be increased by increasing the supported force resulting from reducing the distance between the permanent magnets. The above isolation system requires manual adjustment of the connections of components, while in practice, the components in the vibration isolation system cannot be manually adjusted frequently. To achieve automatic load adjustment, Wen et al. [53] added an electromagnetic constant force mechanism at the end of the QZS isolator to counteract the loads by electromagnetic force. Lu et al. [54] proposed an isolator composed of electromagnetic negative-stiffness and electromagnetic positive-stiffness mechanisms. The QZS isolator can achieve load adjustment by controlling the coil current of the electromagnets. Although electromagnetic control can continuously adjust the loading capacity of the isolation system, the range of the load is limited and requires continuous energy input. Ye et al. [55] and Zhou et al. [56] developed a Multi-layer QZS isolator capable of undertaking different loads at different equilibrium positions. This is composed of a linear-stiffness spring providing positive stiffness and multiple cam-roller mechanisms providing multiple negative-stiffness regions. Thus, in each layer, the QZS property can be realized and different loads can be undertaken. Zeng et al. [57] introduced stair-stepping mechanical metamaterials with a multi-step shape-restoring force curve, based on the programmable characteristics of metamaterials. The height and length of the step can be designed, and each step can undertake a load.

In conclusion, the design methods for a wide-range QZS property or variable loads can be achieved separately. However, there is less research on achieving both requirements simultaneously. In this study, we propose an isolation platform with four Multi-Layer Quasi-Zero-Stiffness (ML-QZS) isolation mounts, appropriate for variable loads. The proposed isolation support is composed of multiple layers of double-ring magnetic mechanisms. Due to the local and nonlinear characteristics of the magnetic force, discontinuous negative-stiffness characteristics can be achieved through a Multi-layer arrangement. Thus, in the proposed vibration isolator, the use of a multiple-point QZS design increases the amplitude range for effective vibration isolation, and the parallel connection of positive spring can generate an ML-QZS design capable of undertaking different loads. The design criterion, therefore, focuses on the coordination of dynamic performances, including the vibration transient attenuation and board frequency displacement transmissibility, both of which depend on all structural parameters. In order to obtain not only the static structural design but also the dynamic performances, the optimization criterions are carried out, and the verification experiment is given. The organization of the paper content is as follows. Section 2 describes the working principle of the ML-QZS with a wide QZS range for various loads and introduces the derivation of restoring force and dynamic stiffness of the ML-QZS isolator. Section 3 establishes the relationship between parameters and dynamic performances, and the bi-objective Pareto optimization criterion is proposed to coordinate vibration transient attenuation and isolation effectiveness across a broad frequency band. The experimental prototype is constructed to verify the theoretical results in Section 4. Lastly, conclusions are given in Section 5.

## 2. Design for ML-QZS Isolation Platform for Variable Loads

### 2.1. Design Method of the ML-QZS Isolation Mounts

**Figure 1:**
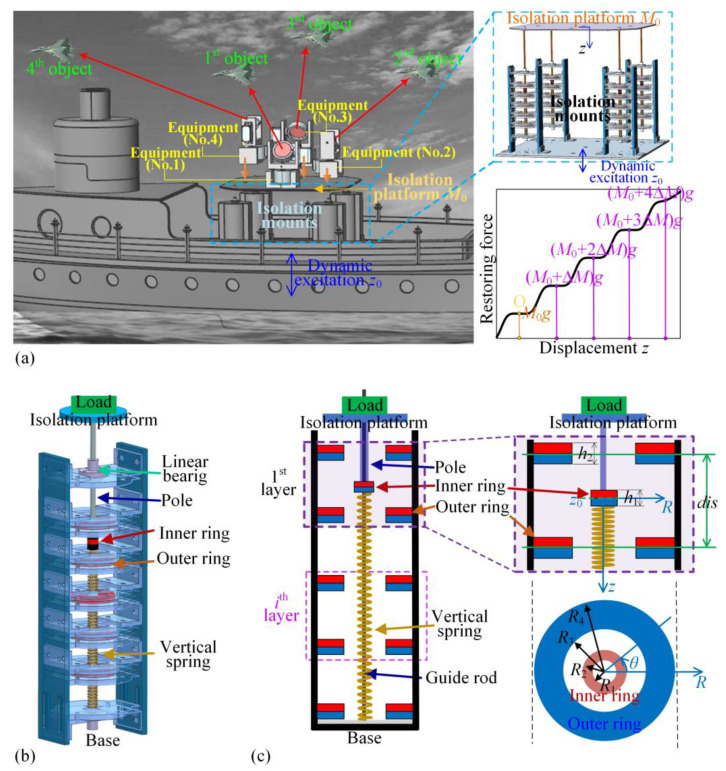
(**a**) An isolation platform applied in low-frequency and large-amplitude vibration environments, required to be appropriate for variable loads for bearing multiple groups of equipment; (**b**) The proposed ML-QZS vibration isolation mount constructed with Multi-layer magnetic rings; (**c**) The structural diagram of the ML-QZS isolation mount and the structural parameters of magnetic rings.

This section provides the design principle of the isolation platform applied for low-frequency and wide-amplitude dynamic excitation. Multiple pieces of equipment can be assembled on the isolation platform, and thus, the platform should have a wide-range QZS property to accommodate variable loads, as shown in Figure 1a. The isolation platform consists of four isolation mounts. In one isolation mount, the Multi-layer magnetic rings for negative stiffness and the vertical spring for positive stiffness are constructed in parallel, as shown in Figure 1b. The load platform is fixed with the inner magnetic ring using the guide rod and multiple outer magnetic rings fixed on the frame. In the magnetic negative-stiffness construction, every magnetic ring has the same magnetization direction and the same axis around the guide rod. The guide rod can only move in the vertical direction under the limitation of a linear bearing. As a result, when the displacement excitation occurs from the bottom, the guide rod, inner ring, and load platform vibrate together vertically.

As shown in Figure 1b, in one isolation mount, the restoring force applied on the isolation platform is the sum of the elastic forces provided by the vertical spring and the Multi-layer magnetic rings. As shown in Figure 1c, the outer double magnetic rings apply magnetic force on the inner magnetic ring. For the magnetic rings, *R*_1_, *R*_2_, *R*_3_, and *R*_4_ are the radius of inner and outer magnetic rings, *h*_1_ and *h*_2_ are the thicknesses of the inner and the outer rings, *B_r_*_1_ and *B_r_*_2_ are the residual magnetic flux density of Neodymium magnetic material, and *μ*_0_ is the air permeability.

**Figure 2:**
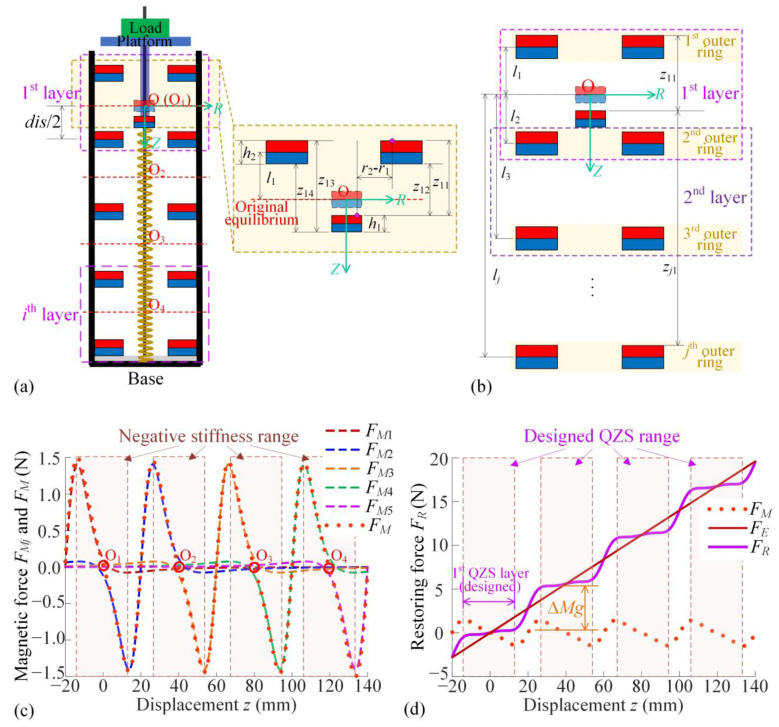
(**a**) The modeling of the proposed ML-QZS vibration isolator and the parameters for a single outer ring; (**b**) the modeling of the restoring force of magnetic rings for Multi-layer outer rings; (**c**) magnetic restoring force applied by each outer ring to the inner ring and their sum; (**d**) restoring force *F_R_* for the ML-QZS property by the combination of *F_M_* and *F_E_*, and the loading capacity in different magnetic layers.

The magnetic force between the inner ring and outer rings is generated only in the vertical direction due to the axially symmetrical configuration, as shown in Figure 2a. According to Ref. [27], using the equivalent magnetic charge method, the magnetic force applied on the inner ring (rigidly connected to the mass) of one layer is given as

(1) $$\begin{array}{l} \;F_{M1}\left(z,l_{j},R_{1},R_{2},R_{3},R_{4},h_{1},h_{2}\right)\\ =\frac{B_{r1}B_{r2}}{4\pi \mu_{0}}\sum_{k=1}^{4}\int_{R_{3}}^{R_{4}}\int_{R_{1}}^{R_{2}}\int_{0}^{2\pi }\int_{0}^{2\pi }\frac{z_{1k}r_{1}r_{2}}{{\left[z_{1k}^{2}+{\left(r_{1}\cos\theta_{1}-r_{2}\cos\theta_{2}\right)}^{2}+{\left(r_{1}\sin\theta_{1}-r_{2}\sin\theta_{2}\right)}^{2}\right]}^{\frac{3}{2}}}d\theta_{1}d\theta_{2}dr_{1}dr_{2} \end{array}$$

where *z*_1*k*_ (*k* = 1, 2, 3, 4) is the distance between the inner and outer ring surfaces as the detail diagram in Figure 2a. For the multiple layers of outer magnetic rings as shown in Figure 2b, the magnetic force applied on the inner magnetic ring is defined as *F_M_*, given as

(2) $$\begin{array}{l} F_{M}(z,R_{1},R_{2},R_{3},R_{4},h_{1},h_{2})=\sum_{j=1}^{i+1}F_{Mj}\left(z,l_{j},R_{1},R_{2},R_{3},R_{4},h_{1},h_{2}\right)\\ \;\;=\frac{B_{r1}B_{r2}}{4\pi \mu_{0}}\sum_{j=1}^{i+1}\sum_{k=1}^{4}\int_{R_{3}}^{R_{4}}\int_{R_{1}}^{R_{2}}\int_{0}^{2\pi }\int_{0}^{2\pi }\frac{z_{jk}r_{1}r_{2}}{{\left[z_{jk}^{2}+{\left(r_{1}\cos\theta_{1}-r_{2}\cos\theta_{2}\right)}^{2}+{\left(r_{1}\sin\theta_{1}-r_{2}\sin\theta_{2}\right)}^{2}\right]}^{\frac{3}{2}}}d\theta_{1}d\theta_{2}dr_{1}dr_{2} \end{array}$$

where *j* denotes the *jth* outer magnetic ring and *i* denotes the *jth* layer. In Equation (2), *z_jk_* is the distance of the surfaces between the inner ring and each outer magnetic ring, as shown in Figure 2b. The first subscript *j* in *z_jk_* indicates the *j*th layer’s outer magnetic ring, and the second subscript *k* indicates the four different surface distances, as shown in Figure 2a. The symbol *z_jk_* (*k* = 1, 2, 3, 4) has the follwoing detailed expression:

(3) $$\begin{array}{l} z_{j1}=z-h_{1}/2-\left(l_{j}+h_{2}/2\right),z_{j2}=z-h_{1}/2-\left(l_{j}-h_{2}/2\right),\\ z_{j3}=z+h_{1}/2-\left(l_{j}+h_{2}/2\right),z_{j4}=z+h_{1}/2-\left(l_{j}-h_{2}/2\right), \end{array}$$

where *l_j_* is the location of the *j*th layer’s outer magnetic ring from the origin vertically in the positive direction of the *Z*-axis. There is a relationship between each *l_j_*, given as *l_j_*_+1_ − *l_j_ = dis* and *l*_1_ *=* −*dis*/2.

The linear spring with positive stiffness *k* is constructed parallel for supporting the load. Considering the restoring force provided by the linear vertical spring as *F_E_* = *kz*, the restoring force of the proposed ML-QZS isolator is defined as *F_R_*, the sum of the magnetic force and the elastic force of the linear spring, given as

(4) $$F_{R}=F_{E}+F_{M}=kz+F_{M}$$

Then, the magnetic forces exerted by each outer magnetic ring *F_Mj_* and by Multi-layer outer magnetic rings *F_M_* are shown in Figure 2c, and the sum restoring force *F_R_* applied to the inner magnetic ring is shown in Figure 2d, with the distance between the double-layer outer rings set at *dis* = 40 mm.

For the original equilibrium fixed at the point O (O_1_), the center of the first layer of double magnetic rings, the dashed lines in red represent the magnetic forces *F*_M1_ for the first layer of the double outer-ring design. Since the outer rings of other layers induce little effect due to the large distance, the red dotted line—representing the sum of the magnetic force acting on the inner ring in the first layer—reveals a wide range for a negative-stiffness property. Similarly, when the inner ring locates the positions at the middle of each double magnetic ring layer as *z* = 0 mm (*z* = 40 mm, *z* = 80 mm, and *z* = 120 mm), the magnetic force has a wide-range negative-stiffness property at these equilibriums. With the positive restoring force provided by the linear vertical spring, the sum restoring force *F_R_* displays step-shape QZS characteristics, as shown in Figure 2d. As the total number *n* of double magnetic ring layers increases, a wider range of load variations can be achieved.

### 2.2. Structural Optimization for Small-Variation Load Step and Wide-Range QZS Property

On one hand, for the Multi-layer double magnetic ring design principle as shown in Figure 2a, the restoring force *F_R_* shown in Figure 2d demonstrates that every step of the restoring force curve can undertake variable loads with the step Δ*Mg*. Taking the center points O*_i_* of each double magnetic ring layer as the equilibrium for QZS vibration isolation, the loading capacity at each layer is given by the sum mass *M_i_*, written as

(5) $$M_{i}=M_{1}+\frac{F_{R}}{g}=M_{1}+\frac{{\left.\left(F_{M}+kz\right)\right|}_{z=(i-1)dis}}{g}=M_{1}+\frac{{\left.\sum_{j=1}^{i}F_{Mj}\right|}_{z=(i-1)dis}+(i-1)kdis}{g}=M_{1}+\left(i-1\right)\Delta M$$

where *M*_1_ is the initial mass, undertaken at the equilibrium in the first double magnetic ring layer at O (O_1_), and Δ*M* is the variable mass undertaken by the vertical spring for each layer. The variation-step load Δ*M* obtained by Equation (5) is given as

(6) $$\Delta M=\frac{{\left.F_{M}\right|}_{z=0}+kdis}{g}$$

On the other hand, from Equation (4), the sum magnetic force *F_M_* depends on both the adjustable structural parameter *dis* and the stiffness coefficient *k* of the vertical positive spring. The two structural parameters are required to satisfy the requirement for a wide-range QZS property around each equilibrium. The stiffness induced by the magnetic field and elastic component, defined as *K_R_*, can be obtained by the following derivation:

(7) $$\begin{array}{l} K_{R}=\frac{dF_{R}(z,R_{1},R_{2},R_{3},R_{4},h_{1},h_{2},dis)}{dz}=k+\frac{dF_{M}(z,R_{1},R_{2},R_{3},R_{4},h_{1},h_{2},dis)}{dz}\\ \;\;\;\;\;=k+\frac{B_{r1}B_{r2}}{4\pi \mu_{0}}\sum_{j=1}^{n+1}\sum_{k=1}^{4}\int_{R_{3}}^{R_{4}}\int_{R_{1}}^{R_{2}}\int_{0}^{2\pi }\int_{0}^{2\pi }\left[\frac{r_{1}\;r_{2}\;}{{\left(z_{jk}^{2}+{\left(r_{1}\cos\theta_{1}-r_{2}\cos\theta_{2}\right)}^{2}+{\left(r_{1}\sin\theta_{1}-r_{2}\sin\theta_{2}\right)}^{2}\right)}^{3/2}}\right.\\ \;\;\;\;\;\;\left.-3\frac{\;z_{jk}^{3}\;r_{1}\;r_{2}\;}{{\left(z_{jk}^{2}+{\left(r_{1}\cos\theta_{1}-r_{2}\cos\theta_{2}\right)}^{2}+{\left(r_{1}\sin\theta_{1}-r_{2}\sin\theta_{2}\right)}^{2}\right)}^{5/2}}\right]d\theta_{1}d\theta_{2}dr_{1}dr_{2} \end{array}$$

In order to obtain the QZS property with static stability at equilibrium, the linear stiffness of the positive spring *k* should satisfy the following condition:

(8) $$k=\left|\min\left({\left.\frac{dF_{M}}{dz}\right|}_{z=0}\right)\right|$$

**Figure A1:**
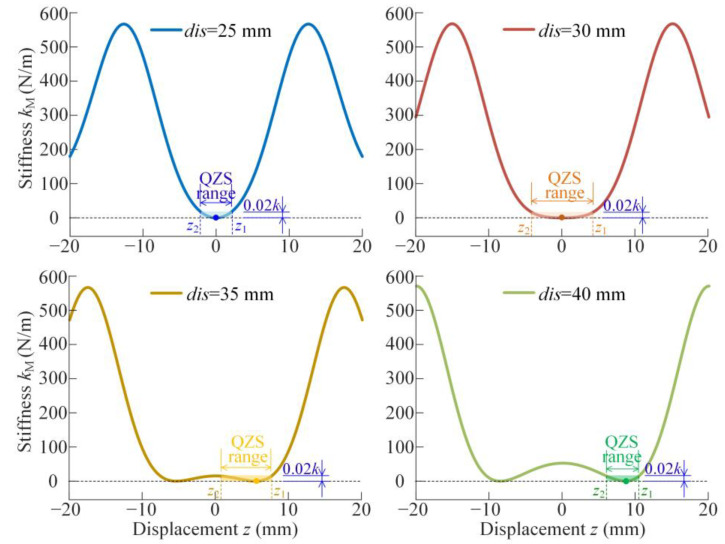
The QZS range in each double-ring layer for *dis* = 25 mm, *dis* = 30 mm, *dis* = 35 mm, and *dis* = 40 mm.

Since the dynamic stiffness of the positive spring is equal to the minimum value of negative stiffness as shown in Equation (8), the QZS property can be realized. Considering the vibration around the first equilibrium O_1_, the equivalent dynamic stiffness *K_R_* for different values of *dis* are shown in Figure A1 in Appendix A. We define the QZS range as *L*_QZS_, the region with equivalent stiffness in [0, 0.02 *k*], expressed by the following condition:

(9) $$L_{\mathrm{QZS}}=\left\|z_{1}-z_{2}\right\|$$

where *z*_1_ and *z*_2_ are obtained by the condition *K_M_* = 0.02 *k* and ordered from large to small value.

According to Equations (2) and (7), the magnetic force *F_M_* and magnetic stiffness *K_M_* are proportional to *B_r_*_1_ and *B_r_*_2_. The QZS length *L_QZS_* is the region with equivalent stiffness in [0, 0.02 *k*], where *k* is the linear stiffness of the positive spring. The linear stiffness of the positive spring *k* is also proportional to *B_r_*_1_ and *B_r_*_2_, according to Equation (8). Therefore, the residual magnetic flux density of Neodymium magnetic material *B_r_* does not affect the quasi-zero-stiffness range. The material and environment’s temperature and humidity only affect *B_r_*_1_ and *B_r_*_2_, so it does not affect the QZS length *L_QZS_*.

**Figure 3:**
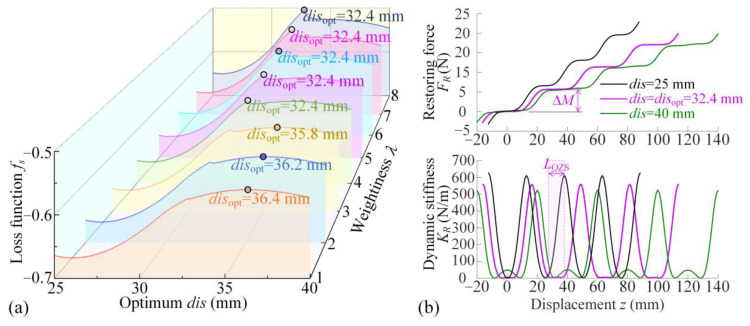
The optimum structural parameter *dis* and the corresponding indices Δ*M* and *L*_QZS_. (**a**) The loss function *f_s_* and the *dis*_opt_ for different weightiness *λ*; (**b**) Two indices Δ*M* and *L*_QZS_ for optimum *dis*_opt_ = 32.4 mm as *λ* = 5, compared to the other values for *dis* = 25 mm and *dis* = 40 mm.

In the structural design of the double-ring system, two indices, including the variation step of load Δ*M* and the range of QZS property *L*_QZS_, have been defined and described. The optimization criterion of the key structural parameter *dis* is given by the loss function *f_s_* as

(10) $$\begin{array}{l} \left\{\left.dis_{\mathrm{opt}}\right|\max\;f_{s}\left(dis\right)=-\Delta M+\lambda L_{\mathrm{QZS}}\right\}\\ s.t.dis\in \left[25\;\mathrm{mm},40\;\mathrm{mm}\right]\; \end{array}$$

where *λ* is the weightiness and Δ*M* and *L*_QZS_ are defined as Eqiations (6) and (9). Figure 3a gives the optimum values *dis*_opt_ for different weightiness *λ* from 1 to 8. As weightiness coefficient *λ* is larger than 4, the range of the QZS property is predominant and the optimum distance of the double magnetic rings is *dis*_opt_ = 32.4 mm. Figure 3b shows that the comparisons of restoring force *F_R_* and dynamic stiffness *K_R_* are among the cases for the optimum structural parameter and two other values as fixing the weightiness *λ* = 5. From the comparison, the variation-step of the load is the smallest and the QZS range is widest for *dis*_opt_.

In summary, the optimization criterion for structural parameter of the double-rings design is proposed for the ML-QZS property with small variation-step of different loads. However, the analysis and results above are based on the restoring force rather than the dynamic performances of the isolation system. Since dynamic characteristics depend on not only structural parameters but also the vibration states, the dynamic performances should be optimized by appropriate structural and damping coefficients, which could be discussed in the following section.

## 3. Optimization for Dynamic Performances

In this section, the dynamic performances of the proposed ML-QZS isolator are analyzed, Based on this, the optimization for dynamic performances is provided. The ML-QZS isolator is designed to be appropriate for variable loads and wide-range QZS vibration isolation, and thus, the dynamic performances, including the vibration isolation, a single-layer wide frequency band, and the transient vibration attenuation between adjacent layers, are coordinated and optimized. Both indices depend on the damping coefficient and the structural design. In this section, the optimization criterion is proposed according to nonlinear vibration analysis.

Substituting Equations (A1)–(A3) into the Lagrange’s equation $\frac{d}{dt}\left(\frac{\text{∂}L}{\text{∂}{\dot{z}}_{r}}\right)\frac{\text{∂}L}{\text{∂}z_{r}}=Q$, where *L* is Lagrange function as *L = T* − *V*, the dynamic equation is given as

(11) $$\left\{\begin{array}{l} M_{i}{\ddot{z}}_{r}+F_{R}\left(z_{r}\right)+c{\dot{z}}_{r}=M_{i}g-M_{i}{\ddot{z}}_{e}\\ z_{r}\left(0\right)=0,{\dot{z}}_{r}\left(0\right)=0 \end{array}\right.$$

For the dynamic Equation (11), the vibration solution contains transient vibration and steady-state vibration. Since we have assembled the initial equilibrium at *z_r_* = 0 and the variation of load is Δ*M* for one time, the complete solution contains transient vibration, occurring between the adjacent layer, and steady-state vibration, occurring in one layer. For one layer, the dynamic equation around each equilibrium is given as shown in Equation (A4) in Appendix B.

### 3.1. Bifurcation Condition for Single-Steady State and Displacement Transmissibility Curve

For the dynamic model, as shown in Equations (11) and (A4), in order to solve the steady states in one double-ring layer, we assume that Δ*M* = 0 and apply the Averaging Method. Then, the derivation process for steady states is shown in Appendix B. From Equation (A6), which is the differential equation of the amplitude and phase, in one double-ring layer, the following conditions should be satisfied for steady-state response:

(12) $$\left\{\begin{array}{l} \dot{a}=\frac{1}{2\pi \omega }\left[Y\left(a,\omega \right)-F_{isp}\sin\theta \right]=0\\ \dot{\theta }=\frac{-1}{2\pi \omega a}\left[P\left(a,\omega \right)+F_{isp}\cos\theta \right]=0 \end{array}\right.$$

In Equation (12), the expressions of *Y* (*a*,*ω*), *P* (*a*,*ω*), and *F_isp_* are given in Appendix A. According to the sum of squares formula of trigonometric functions, it has the following equation:

(13) $$Y{\left(a,\omega \right)}^{2}+P{\left(a,\omega \right)}^{2}-F_{isp}^{2}=0$$

Equation (13) gives the relationship between frequency and vibration amplitude. The solution from Equation (13) is obtained as *a*_0_, and the solution for phase is *θ*_0_, given as

(14) $$\left\{\begin{array}{l} \sin\theta_{0}=Y\left(a_{0},\omega \right)/F_{isp}\\ \cos\theta_{0}=-P\left(a_{0},\omega \right)/F_{isp} \end{array}\right.$$

Then, the transmissibility of the ML-QZS isolator is obtained from the ratio of the maximum response amplitude of the load and excitation amplitude of the exciter, and is given as

(15) $$T_{d}=10\mathrm{lg}\left(\frac{\max\left(z_{d}\right)}{z_{0}}\right)=10\mathrm{lg}\left(\frac{\sqrt{a_{0}^{2}+z_{0}^{2}+2a_{0}z_{0}\cos\theta_{0}}}{z_{0}}\right)$$

Due to the nonlinearity induced by the magnetic force, the multi-steady states result from bifurcation, which causes the jumping phenomenon of vibration.

Based on the eigenvalues of Jacobian Matrix **J** in Equation (A10), the differential equation for the perturbation variables around the single steady state (*a*_0_, *θ*_0_) is derived, allowing the determination of critical parameters for both single- and multi-steady states. The eigenvalues *λ* of the Jacobian Matrix **J** can be obtained by solving the equation *λ^2^* – tr (**J**)*λ +* det (J) *=* 0. According to the Routh–Hurwitz criterion for the stability of the steady states, the state is asymptotic stability when tr (**J**) *<* 0 and det (**J**) *>* 0. In Equation (A10), as tr (**J**) is negative, the condition det (**J**) *>* 0 corresponds to the stable steady state. Because the multi-steady states include both stable and unstable solutions, the boundary separating the single steady-state range from the multi-steady-state range is given by the condition det (**J**) *=* 0, written as

(16) $$\det\left(J\right)=c^{2}+\frac{\left[M_{i}a_{0}\omega^{2}-F_{R}\left(a_{0},\theta_{0}\right)\right]\left[-M_{i}\omega^{2}+K_{R}\left(a_{0},\theta_{0}\right)\right]}{a_{0}\omega^{2}}=0$$

**Figure 4:**
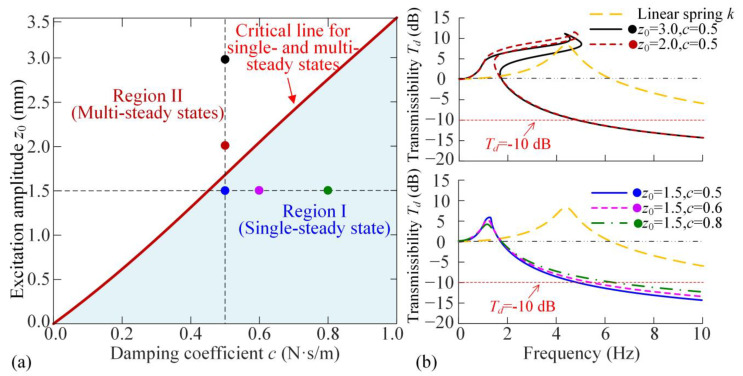
(**a**) Comparison of the two constructions for the single outer ring and double outer rings; (**b**) critical excitation amplitude for single and multiple steady-state responses for different excitation amplitudes *z*_0_ and damping coefficients *c*.

**Figure A2:**
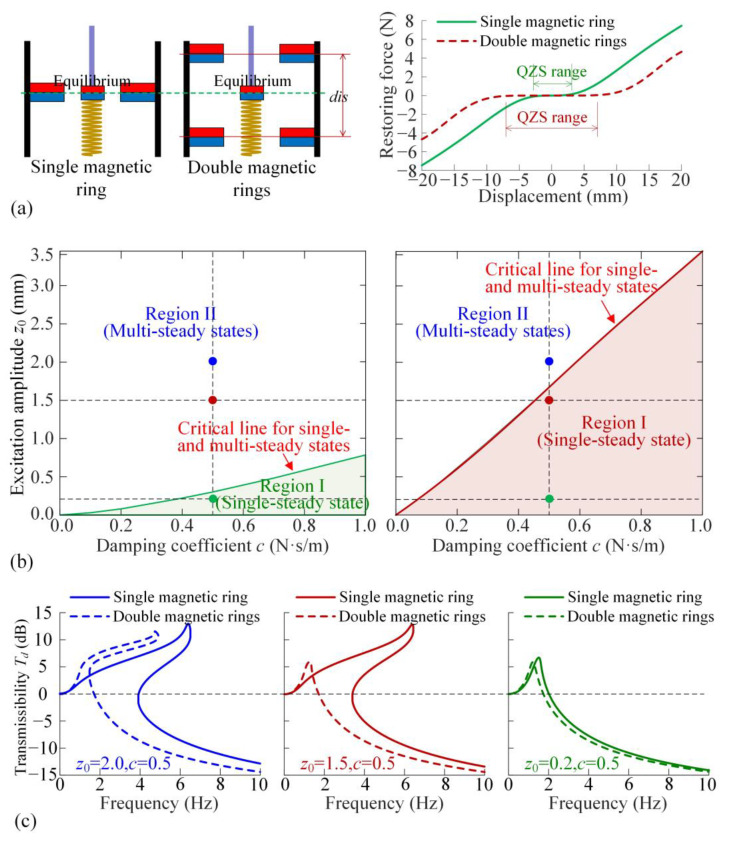
(a) Comparison of single-ring configurations and multi-ring configurations and their restoring forces; (b) Critical excitation amplitude *z*_0_ for different *dis*; (c) The transmissibility for different excitation amplitude *z*_0_.

From Equation (16), the boundary of excitation amplitude *z*_0_ and damping coefficient *c* for single- or multi-steady states around the resonance is shown in Figure 4a for *dis*_opt_ = 32.4 mm. In Region I, there is only a single-steady state around the resonance, while in Region II, the multi-steady states occur. Increasing the damping coefficient c would raise the excitation amplitudes. Figure 4b shows the amplitude-frequency curves with the double-ring design for different parameters fixed in Region I and II. By comparison with the linear spring, the proposed QZS design by double magnetic rings has much lower effective isolation initial frequency and achieves the low-frequency isolation, which displays the advantages of the proposed nonlinearity. The system is stable if the excitation amplitude does not exceed the critical excitation amplitude. However, for the existence of multi-steady states, the vibration amplitude would jump to a large amplitude, which is the disadvantage of nonlinearity. Fortunately, as shown in Figure A2 in Appendix C, with the comparison of Region I between single magnetic ring (proposed in Ref. [17]) and double magnetic-ring designs, the proposed double-ring QZS isolator has a larger range for excitation amplitude for the single-steady state. Additionally, more comparisons of the isolator’s performance for different QZS isolators are shown in Appendix C, which verifies the advantages of the double-ring design principle for the wide-amplitude QZS property.

In addition, from the displacement transmissibility curves in Region I for different damping coefficients, as shown in Figure 4b, increasing the damping coefficient c would raise the displacement transmissibility in the high-frequency band. To address the requirement that the vibration is reduced to 10%, the transmissibility *T_d_* should equal to −10 dB. The corresponding excitation frequency is around 6 Hz. Thus, we introduce the first index, which is the transmissibility at *ω* = 6 Hz, to evaluate the effectiveness of the vibration isolation, given as

(17) $$T_{e}={\left.T_{d}\right|}_{\omega =6\;\mathrm{Hz}}$$

### 3.2. Transient Vibration Spanning to the Next Layer

For the ML design of the proposed ML-QZS isolator, as the load increases, when the load drops to the next layer, the transient response is a free vibration. The transient response for variable loads can be solved using the following equation as

(18) $$\left\{\begin{array}{l} M_{i}{\ddot{z}}_{r}^{\left(i\right)}+F_{R}\left(z_{r}^{\left(i\right)}\right)+c{\dot{z}}_{r}^{\left(i\right)}=-\Delta Mg\delta \left(t=0\right)\\ z_{r}^{\left(i\right)}\left(0\right)=0,{\dot{z}}_{r}^{\left(i\right)}\left(0\right)=0 \end{array}\right.$$

According to Equation (18), the proposed ML-QZS isolator is only applicable to static loads. The variable load can only be an integer multiple of Δ*M*, not a real-time changing load.

Substituting $z_{r}^{\left(i+1\right)}=z_{r}^{\left(i\right)}+\mathrm{dis}$ into Equation (18) and considering $F_{R}\left(\mathrm{dis}\right)=-\mathrm{Mg}$, the nonhomogeneous equation is transformed into a homogeneous equation as

(19) $$\left\{\begin{array}{l} M_{i}{\ddot{z}}_{r}^{\left(i+1\right)}+F_{R}\left(z_{r}^{\left(i+1\right)}\right)+c{\dot{z}}_{r}^{\left(i+1\right)}=0\\ z_{r}^{\left(i+1\right)}\left(0\right)=dis,{\dot{z}}_{r}^{\left(i+1\right)}\left(0\right)=0 \end{array}\right.$$

**Figure 5:**
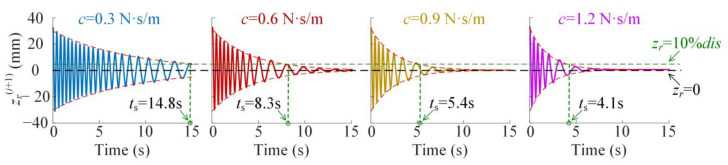
The transient response for different damping coefficients *c* as *dis* = 32.4 mm.

According to Ref. [11], the envelope curve of the transient vibration can be expressed as

(20) $$z_{r}^{\left(i+1\right)}=dis*\exp\left[-b\left(a\right)t\right]$$

where *b*(a) is the attenuation function coupled with the transient vibration amplitude. The transient time *t_s_* is defined as the time when the amplitude of *z_r_* is reduced to less than 10% of the initial motion *dis*, given as

(21) $$t_{s}=-\log\left(0.1\right)/b\left(0.1dis\right)$$

which is shown in Figure 5 for different values of *c*.

As shown in Figure 5, the envelope curves described by Equation (21) can obtain the effective vibration transient time, which obviously decreases with the increase of damping coefficient *c*. However, it is known that larger damping would worsen the isolation effectiveness in the high-frequency band. Thus, the optimization criterion for coordination of transient vibration attenuation and isolation effectiveness is needed in the high-frequency band.

### 3.3. Optimization Criterion for Dynamic Performances

**Figure A3:**
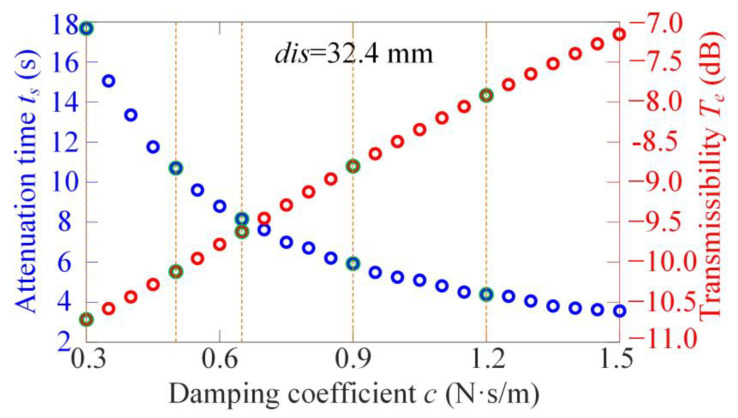
Vibration transient time from the upper layer to the next layer and the displacement transmissibility at a high frequency as *ω* = 6 Hz for different damping coefficients *c*.

**Figure A4:**
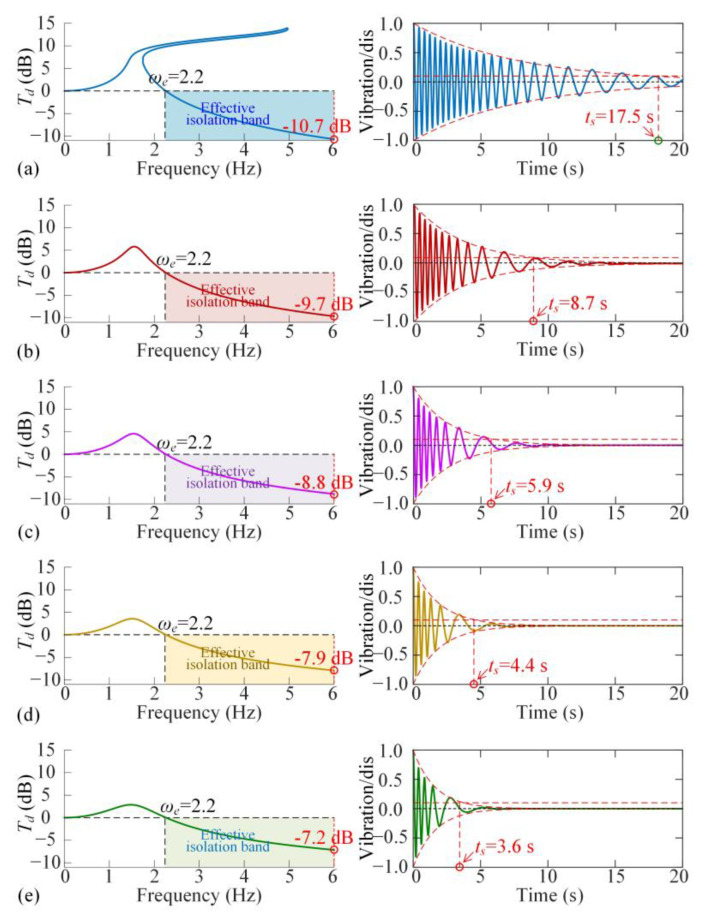
Transient time and transmissibility for different values of damping coefficient *c*, as (**a**) *c* = 0.3; (**b**) *c* = 0.45; (**c**) *c* = 0.64; (**d**) *c* = 0.9; (**e**) *c* = 1.2.

**Figure A5:**
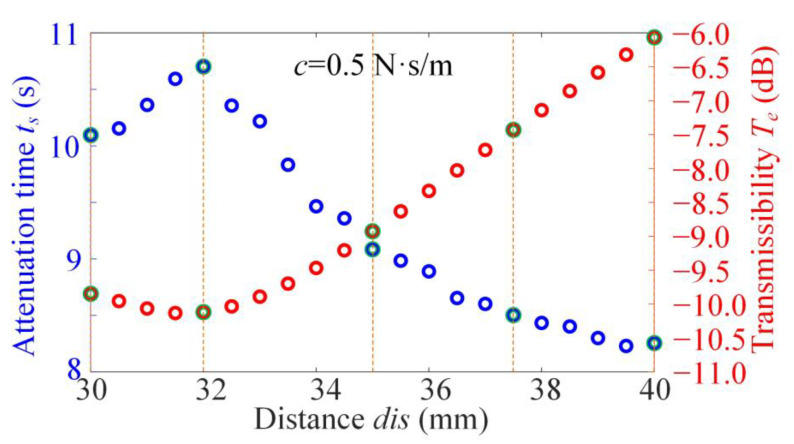
Vibration transient time from the upper layer to the next layer and the displacement transmissibility at a high frequency as *ω* = 6 Hz for different distance *dis*.

**Figure A6:**
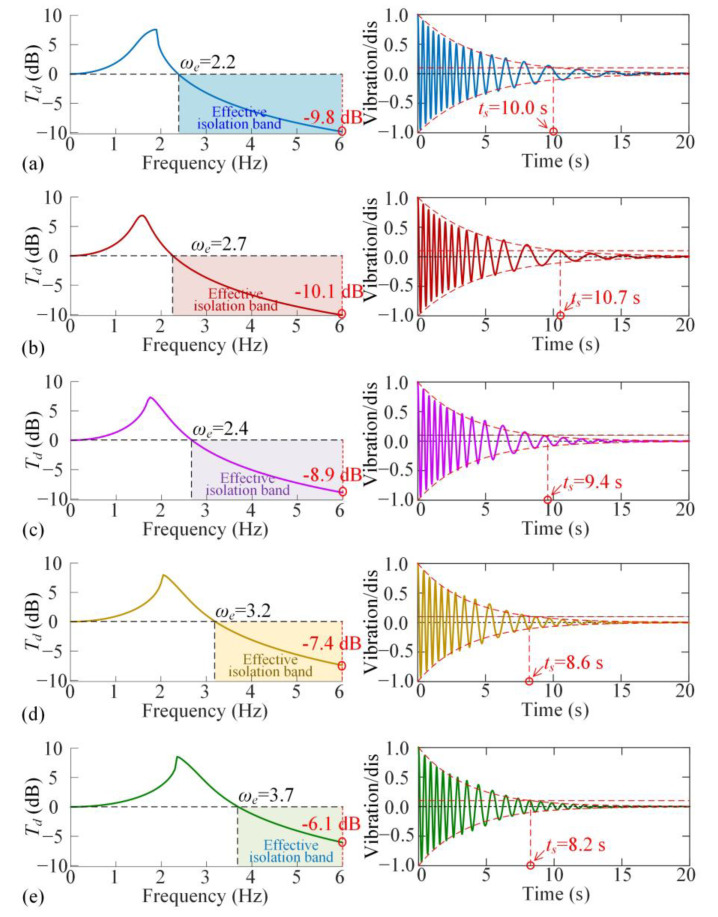
Transient time and transmissibility for different parameter *dis*, as (**a**) *dis* = 30.0 mm; (**b**) *dis* = 32.0 mm; (**c**) *dis* = 35.0 mm; (**d**) *dis* = 37.5 mm; (**e**) *dis* = 40.0 mm.

According to the analysis in Section 3.2, the vibration isolation band and vibration attenuation are obtained using the Averaging Method. The two indices, including transient time and isolation effectiveness in the high-frequency band, mainly depend on the damping coefficient *c*. According to the practical needs, it is required to simultaneously reduce the transient time and the displacement transmissibility. As shown in Figure A3 and Figure A4 in Appendix D and Figure A5 and Figure A6 in Appendix E, the two objectives are contradictory and incompatible. To coordinate the vibration transient time for variable loads and transmissibility, the multi-objective optimization criterion is proposed as follows:

(22) $$\begin{array}{l} \min F(\mu )=\left\{t_{s}(\mu ),T_{e}(\mu )\right\}\\ s.t.\mu =(c,dis)\in \mathbb{P}\\ \mathbb{P}=\{0.3\;N\cdot s\cdot m^{-1}\leq c\leq 1.2\;N\cdot s\cdot m^{-1},30\;\mathrm{mm}\leq dis\leq 35\;\mathrm{mm}\} \end{array}$$

where **μ** denotes the optimization parameter vector and P is the feasible domain of structural parameters *c* and *dis*. In function *F*(**μ**), *t*_s_ is the transient time for variable loads; *T_e_* is the value of displacement transmissibility at 6 Hz. Although the optimum value of *dis* is *dis*_opt_ = 32.4 mm obtained using static analysis in Section 2.2, we vary it in the range of 30 mm to 35 mm to coordinate the two dynamic indices.

**Figure 6:**
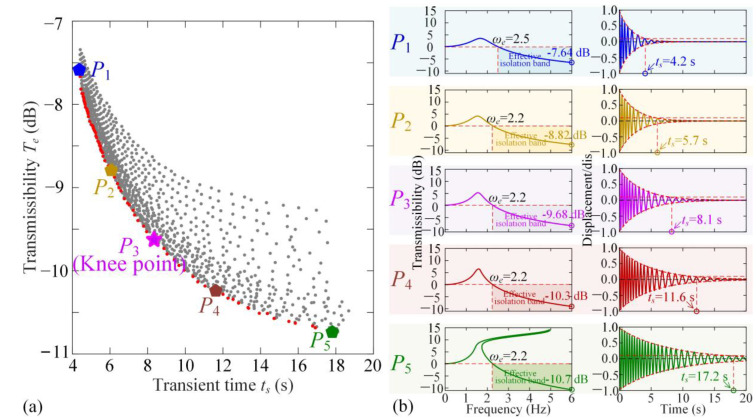
The Pareto optimization for coordinating vibration attention time *t_s_* and displacement transmissibility *T_e_*. (**a**) The Pareto front points and Knee point in the objective space for transient time and transmissibility; (**b**) at the Pareto front points, *P*_1_, *P*_2_, *P*_4_, *P*_5_, and the Knee point *P*_3_, the transmissibility curves, and the values at 6 Hz, transient responses, and the attenuation times.

Figure 6 shows the feasible index solution that *t*_s_ and $T_{\omega_{a}}$ vary for different values of *c* and *dis*, marked with grey points. According to Equation (22), if there is no value of $\overset{⌢}{\mu }=(\overset{⌢}{c},\overset{⏜}{dis})$, so that *F*(**μ**) < $F(\overset{⌢}{\mu })$, then $F(\overset{⌢}{\mu })$ is the Pareto index solution and the parameter set $\overset{⌢}{\mu }=(\overset{⌢}{c},\overset{⏜}{dis})$ is called the Pareto front, as shown with red points in Figure 6a. All points for the Pareto front meet the design requirement for dynamic performance optimization. However, the variations of the two dynamic objectives, transient time *t_s_* and transmissibility at high-frequency *T_e_*, are contradictory, so the Knee Point is selected from all Pareto front points for coordination of the two indices. Figure 6b shows the transmissibility curve and the vibration transient at five Pareto front points from *P*_1_ to *P*_5_. Defining the Knee Point as the one with the longest distance from the line across the two edge points *P*_1_ to *P*_5_, we figure out that *P*_3_ is the optimization indices for dynamic performances. At the Knee Point *P*_3_, where *dis* = 32.2 mm and *c* = 0.64, the displacement transmissibility at 6 Hz is −9.68 dB, indicating that the vibration excitation is reduced to about 10% under continuous base excitation. Additionally, the vibration is effectively attenuated at 8.1 s as the system transitions from the upper layer to the lower one as the loading increases Δ*M*.

## 4. Isolation Platform and Experimental Verification for Structural Optimization

In this section, the prototype of the ML-QZS isolator platform is constructed, and the experiment processes are carried out to show the optimum design for the coordination of dynamic performances.

### 4.1. Experimental Prototype for One ML-QZS Isolation Mount

**Figure 7:**
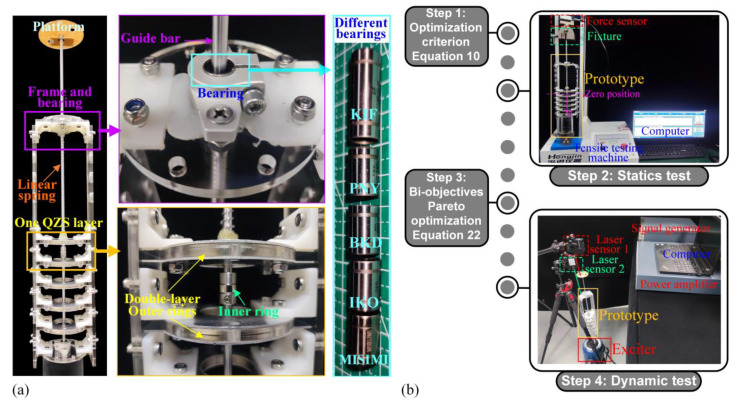
(**a**) Experimental prototype of one isolation mount with the ML-QZS design. In one QZS layer, there are double magnetic rings and different linear bearings are applied in the system at the connection between frame and guide bar. (**b**) Experimental processes of the proposed single ML-QZS isolation mount (four steps).

The experimental prototype for one ML-QZS isolation leg is shown in Figure 7a. In one leg, the inner magnetic ring is connected to the isolation platform with a rigid guide bar, supported by a linear spring in vertical direction. A linear bearing is connected between the frame and the guide bar (fixed with the platform). In one QZS layer, the double-layer outer magnetic rings are configured for the wide-amplitude QZS property as shown in the analysis in Section 2.2. In the double magnetic layer, the inner and outer diameters of the inner magnet are 5 mm and 10 mm; the inner and outer diameters of the two outer magnetic rings are 30 mm and 50 mm. The residual magnetic flux density of the magnetic rings is 0.9 T. Five standard linear bearings are utilized in the comparison of dynamic performances, with the types of KIF-LM-5LUU, PNY-LM-5UU, BKD-LM-5UU, IKO-LM-5UU, and MISIMI-LM5P. Through simple parametric regression testing, the damping coefficients for KIF, PNY, BKD, IKO, and MISIMI, are 0.35 N·s·m^−1^, 0.44 N·s·m^−1^, 0.65 N·s·m^−1^, 0.89 N·s·m^−1^, and 1.21 N·s·m^−1^, respectively.

The experimental processes are constructed as shown in Figure 7b, typically consisting of four Steps. Step 1 is the structural design of the double-layer magnetic rings for the widest range for the QZS property and the smallest variation for different loads according to the optimization criterion (10). Step 2 is the static testing verification for the variation loading and range for the QZS property, which can provide the prediction for the excitation amplitude range for the single-steady state and avoid the jumping phenomenon. Step 3 involves optimizing the linear bearing and structure using the bio-objective optimization criterion (23). Step 4 is the dynamic testing verification for the dynamic performances including the transient vibration attenuation at the adjacent layer for increasing the loading and the vibration isolation effectiveness in one double-ring QZS layer. In the dynamic testing, the base excitation is generated by the exciter (VE-5110, Yiheng Technology Co., Ltd., Hangzhou, Zhejiang, China), which is driven by a signal generator (ECON-MI8008, Yiheng Technology Co., Ltd., Hangzhou, Zhejiang, China) with a power amplifier (ECON-H181A, Yiheng Technology Co., Ltd., Hangzhou, Zhejiang, China). The vibration frequency and amplitude of the exciter are controlled using the software (TestEngineX 4.2.40) of the signal generator. In this paper, the vibration amplitude is set to 1.2 mm for all dynamic tests. Two laser sensors (KathMaticKVD-4525L, KathMaticKVD-4525S, KathMatic Technology Co., Ltd, Nanjing, Jiangsu, China) are placed to estimate the absolute vibrations of the base and the isolation platform in the time domain, respectively. Then, the data, measured using laser sensors, are collected and sent to the computer, and analyzed using the signal processing software to obtain the amplitude.

### 4.2. Experiment Results

**Figure 8:**
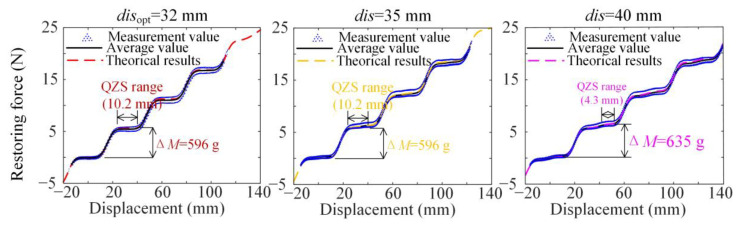
The comparison of variation-steps of loading Δ*M*g and QZS range among *dis*_opt_ = 32 mm, *dis* = 35 mm, and *dis* = 40 mm based on theorical results and the unidirectional tensile testing of one ML-QZS isolation mount.

According to the optimization criterion of the structural design in Equation (10), the vertical positive-spring stiffness is *k* = 0.196 N·mm^−1^ and the optimum value of *dis* is *dis*_opt_ = 32.0 mm. In order to show the comparison of the QZS range and loading variation for different values of structural parameter *dis*, we construct other three prototypes with *dis*_opt_ = 32 mm, *dis* = 35 mm, and *dis* = 40 mm. Then, through quasi-static testing using the tensile testing machine, the restoring forces *F_R_* for different *dis*_opt_ are obtained as shown in Figure 8. For the *dis*_opt_, the initial load mass is *M*_0_ = 238.6 g, with 10.2 mm QZS range and variation load Δ*M* = 596 g. While for the cases *dis* = 35 mm and *dis* = 40 mm, the loading variations are both larger and the QZS ranges are both narrower compared to the optimum case.

**Figure 9:**
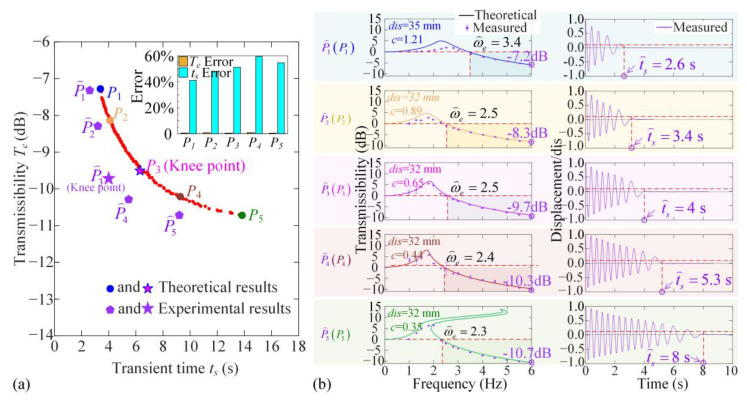
(**a**) Pareto front points based on theoretical and experimental results and the point closest to the Knee point figured out among them and the error; (**b**) the comparison of displacement transmissibility frequency curves and vibration attenuations obtained through experiments at the five Pareto front points (including the ${\overset{⌢}{P}}_{3}$ closest to the Knee point), corresponding to five groups of parameters *dis* and damping coefficients *c*.

Based on the above experimental results from tensile testing, the main structural parameter *dis* is chosen at the optimum value *dis*_opt_. After fixing the structural parameter *dis*, the Pareto optimization, as shown in Equation (22), is carried out to determine the optimum damping coefficient for dynamic performances, including vibration attenuation and isolation effectiveness, as shown in Figure 9. As shown in Figure 9a, the Pareto front points based on theoretical and experimental results are given, represented by point *P_i_* and ${\overset{⌢}{P}}_{i}$, respectively. In the experimental analysis, five Pareto front points are shown with the damping coefficients for the five different linear bearings shown in Figure 7a. For the linear bearing with the model number BKD-LM-5UU, the damping coefficient *c* = 0.65 N·s·m^−1^ is the closest value to the Knee point. Then, the experimental results for displacement frequency and transient attenuation for the five prototypes with structural parameters and damping coefficients at the five Pareto front points are shown in Figure 9b.

As shown in Figure 8, the theoretical restoring force is not completely consistent with the measured value, which is caused by nonlinear damping. As shown in Figure 9a, the transient response time in the optimization test is significantly smaller than the calculation result, but has no effect on the steady-state response results and the parameter of optimization result.

By comparing the displacement transmissibility frequency curves and vibration transient attenuations with fixed parameters among the five Pareto front points, the damping coefficient at ${\overset{⌢}{P}}_{3}$ can coordinate the two dynamic indices. For structural parameter *dis*_opt_ = 32 mm and BKD-LM-5UU with *c* = 0.65 N·s·m^−1^≈*c*_opt_, the effective vibration isolation begins at 2.5 Hz, the displacement transmissibility reaches to −10 dB at 6 Hz, and the transient vibration is attenuated in 4 s. The corresponding videos are shown in Video S1. Therefore, the proposed bi-objective optimization can coordinate multiple dynamic performances, and the proposed ML-QZS isolation mount has low-frequency isolation effectiveness for variable loads.

### 4.3. Isolation Platform with ML-QZS Property for Variable Loads

**Figure 10:**
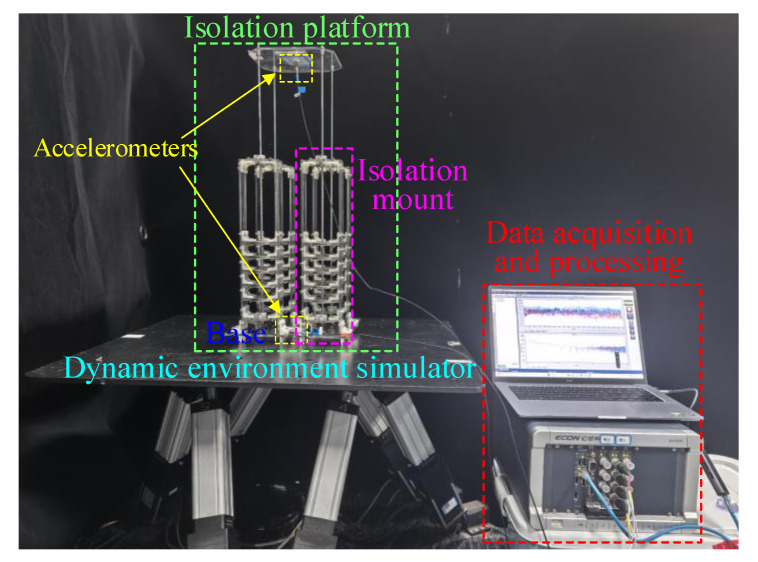
The isolation platform with four ML-QZS isolation mounts with optimization design and damping coefficient.

The isolation platform, as displayed in Figure 10, is constructed using four isolation mounts with the ML-QZS property design for *dis*_opt_ = 32 mm and BKD-LM-5UU with *c* = 0.65 N·s·m^−1^≈*c*_opt_. When manufacturing the isolation platform, each leg’s damping is identified to control parameter consistency and reduce error. In the dynamic experimental prototype, as shown in Figure 10, a six-DOF motion platform simulates the low-frequency excitation (NJQK-STEWRT, Nanjing All Controller Electronic Technology Co., Ltd. Nanjing, Jiangsu, China), two accelerometers (DYTRAN-3023A, Dytran Instruments, Inc., Chatsworth, CA, USA) are fixed on the isolation platform and the base, and the signals are sent to the data acquisition and processing system. The absolute vibration displacement is obtained by integrating the acceleration data twice in time, and then the amplitude is obtained.

**Figure 11:**
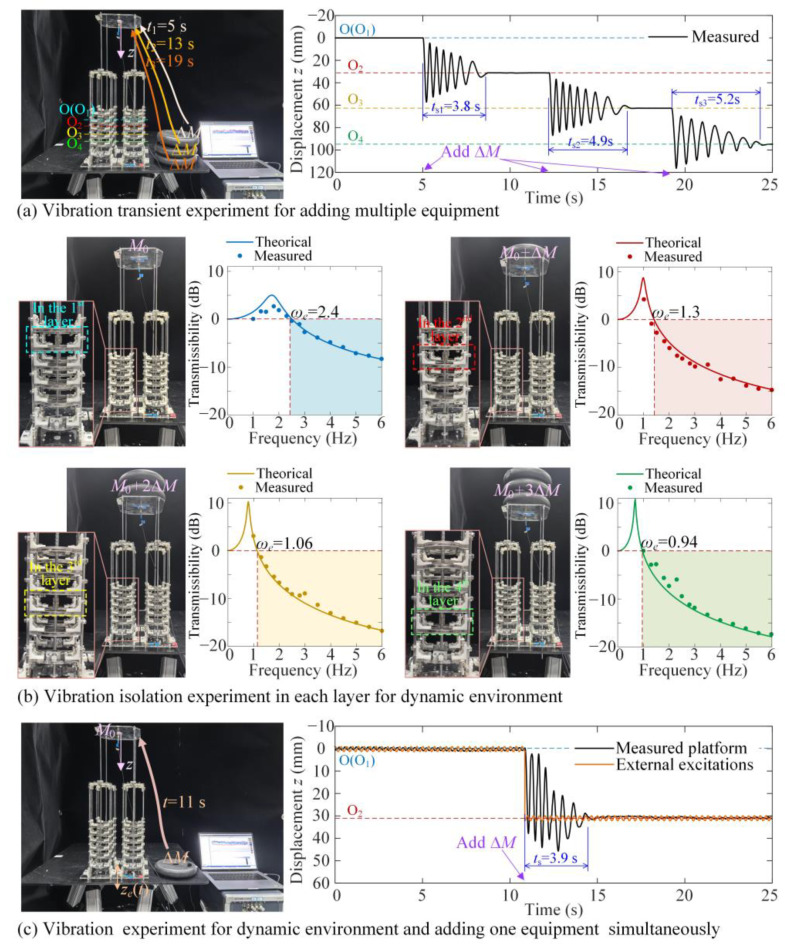
The dynamic experimental results. (**a**) The vibration transient experiment for adding multiple equipment; (**b**) the vibration isolation results in each layer for different loads for dynamic environments; (**c**) the vibration performance for simultaneous application in dynamic environments and variable loads, with an excitation amplitude of 1.2 mm and a vibration frequency a 3 Hz.

In the dynamic experiment for the isolation platform with optimization structural parameters, the isolation performances are demonstrated by three experiments as shown in Figure 11. The first experiment is to show the vibration attenuation effect for adding multiple equipment, the second is to show the isolation effect in different layers for low-frequency excitation, and the third is to show the vibration suppression effect for low-frequency excitation and adding load simultaneously.

As shown in Figure 11a, vibration transient attenuation times for adding the loads as multiple equipment are estimated. We apply the load Δ*Mg* at *t* = 5 s, *t* = 13 s, and *t* = 19 s, and the corresponding theoretical equation for the dynamic performance can be given as

(23) $$\left\{\begin{array}{l} M_{0}\ddot{z}+F_{R}\left(z\right)+c\dot{z}=\Delta Mg{\left.\delta \left(t\right)\right|}_{t=5s}+\Delta Mg{\left.\delta \left(t\right)\right|}_{t=13s}+\Delta Mg{\left.\delta \left(t\right)\right|}_{t=19s}\\ z\left(0\right)=0,\dot{z}\left(0\right)=0 \end{array}\right.$$

where the load was calibrated before the experiment, the variable load for one mount is 596 g.

For the experimental results, the attenuation times for vibration transients from the first layer to the second, third, and fourth layers are *t_s_*_1_ = 3.8 s, *t_s_*_2_ = 4.9 s, and *t_s_*_3_ = 5.2 s, respectively, which reveals that the vibration can be effectively eliminated with the optimum damper chosen from the five model numbers. The corresponding videos are shown in Video S2.

As shown in Figure 11b, the vibration isolation in different layers with different loads are shown. We apply the excitation with amplitude *z*_0_ = 1.2 mm and with frequency *ω* from 1 Hz to 6 Hz. The corresponding theoretical equation for dynamic performance is given as

(24) $$\left\{\begin{array}{l} M_{i}{\ddot{z}}_{r}+F_{R}\left(z_{r}\right)+c{\dot{z}}_{r}=M_{i}g-M_{i}{\ddot{z}}_{e}=M_{i}g+M_{i}z_{0}\omega^{2}\cos\omega t\\ z_{r}\left(0\right)=0,{\dot{z}}_{r}\left(0\right)=0 \end{array}\right.$$

where the definition of *M_i_* is given by Equation (5). The displacement transmissivity *T* for the theoretical analysis is obtained using Equation (15). As shown in Figure 11b, the effect isolation band begins from *ω_e_* = 2.5 Hz, *ω_e_* = 1.3 Hz, *ω_e_* = 1.06 Hz, and *ω_e_* = 0.94 Hz in the first, second, third, and fourth layers with the excitation amplitude *z*_0_ = 1.2 mm. The theoretical results are consistent with the experimental results, proving that the conclusion regarding the system’s stability is effective in the theoretical study. Due to the increase in loading, the frequency band for the subjacent layer becomes wider. Thus, the low-frequency isolation for different loads can be realized. The corresponding videos are shown in Video S2.

Finally, as shown in Figure 11c, the vibration suppression effectiveness when simultaneously applying low-frequency base excitation and variable loads demonstrated. As the base excitation *z*_0_cos *ωt* with *z*_0_ = 1.2 mm and *ω* = 3 Hz, for *t* ranging from 0 s to 11 s, the absolute vibration amplitude of the isolation platform is 50% smaller than *z*_0_, as verified by the displacement transmissibility curves shown in Figure 11b as T = −3 dB. When applying the Δ*Mg* at *t* = 10.5 s, the vibration equilibrium drops from O_1_ to O_2_ in the second layer. The vibration attenuation time is *t_s_* = 3.9 s and the vibration amplitude of the isolation platform is smaller than the excitation amplitude as T = −10 dB for *t* from 14.5 s. The corresponding videos are shown in Video S2.

Therefore, the dynamic performances shown in Figure 11 demonstrate that the proposed isolation platform is suitable for low-frequency dynamic environment with variable loads due to the ML-QZS design. By optimizing the structural parameters and damping coefficient, low-frequency isolation effectiveness and rapid vibration attenuation can be realized.

Table 1 shows the performance comparison for different vibration isolators. Since the structure size has a great influence on the QZS range, we take the ratio of the QZS range and the parameters of structure design as the evaluation index. The starting vibration isolation frequency is chosen as the second evaluation index to evaluate the first-order stiffness of the QZS system at the equilibrium point.

The comparison results show that the proposed quasi-zero-stiffness isolator not only has advantages in the QZS range and the starting vibration isolation frequency range but also can adapt to variable loads.

## 5. Conclusions

This study provides the design principle of an ML-QZS vibration isolation platform, constructed using multiple magnetic rings and positive-stiffness spring. The proposed isolation platform is applicable for low-frequency and large-amplitude dynamic excitation with variable loads. The main results are as follows.

a. We propose the optimization criterion of the structure for the ML-QZS vibration isolation platform applied in the low-frequency and wide-amplitude dynamic environments. The wide-range negative-stiffness property is realized using multiple magnetic rings design, which breaks the locality of the QZS property around equilibrium in traditional triple-springs design.

b. Two dynamic performances, including vibration attenuation time and isolation effectiveness in a broad frequency band, are considered for obtaining the optimum structure and damper. Considering that the variations of the two dynamic performances are contradictory, the Pareto optimization criterion is given for the coordination of different indices.

c. Different groups of experimental prototypes are constructed for comparison. The experimental results not only verify the theoretical results and design principle for the ML-QZS property but also verify the two optimization criteria proposed in this study. The isolation platform is successfully applied in low-frequency excitations with variable loads.

In summary, based on the design principle proposed, the proposed ML-QZS vibration platform has remarkable potential applications in ocean dynamic environments for assembly of variable equipment mass. But the proposed vibration isolation platform is only suitable for integer multiples of variable loads. So, in the future, the quasi-zero-stiffness vibration isolator for adaptive stepless, variable loads will be realized by controlling electromagnetic force.

## Figures and Tables

**Table 1:** The comparison of isolator’s performance for different isolators.

Vibration Isolator	QZS Range/Design Parameter (*k_QZS_* < 0.02 *k_s_*)	$\mathrm{Starting}\;\mathrm{Isolation}\;\mathrm{Frequency}\omega_{e}$(Hz)	Vibrable Load
Linear spring damping isolator	0	6.5	Yes
QZS with triple nonlinear spring [43]	0.2	3.0	No
QZS with single magnetic ring [17]	0.16	3.0	No
QZS with X-shaped structure [28]	0.105	3.5	No
QZS with electromagnetic constant force mechanism [53]	0.112	2.5	Yes
QZS with dual electromagnetic nonlinear stiffness mechanisms [54]	0.250	3.5	Yes
QZS with Multi-layer cam-roller [55]	0.5	3.6	Yes
QZS with mechanical metamaterials [57]	0.08	17.6	Yes
The proposed ML-QZS isolator	0.32	2.5	Yes

## Data Availability

Data will be made available on request.

## Supplementary Materials

The following supporting information can be downloaded at: https://www.mdpi.com/article/10.3390/ma18071676/s1, Video S1: Media Extension I; Video S2: Media Extension II.

## Appendix A

Appendix A shows the effect of parameter dis on the nonlinear stiffness *K_R_* and the definition of width for QZS property *L*_QZS_.

According to Equation (7), considering the vibration around the equilibrium in the first double-ring layer, the nonlinear stiffness *K_R_* for different values of *dis* is shown in Figure A1.

Since the outer double magnetic rings can realize negative-stiffness property, with the positive stiffness of the vertical spring, the QZS property can be realized, and the region width is shown in Figure A1. However, as shown in the stiffness curves in Figure A1, as the distance *dis* increases from 25 mm to 40 mm, the stiffness at equilibrium is far away from zero in the designed QZS range, especially for *dis* larger than 35 mm. For the two cases *dis* = 35 mm and *dis* = 40 mm, the QZS property range is less than the case *dis* = 30 mm. Then, the QZS property range is defined as the region with equivalent stiffness in [0, 0.02 *k*], as shown in Equation (9).

## Appendix B

Appendix B shows the solving process of the dynamic behaviors of the ML-QZS isolator using the Averaging Method and the critical condition for vibration jumping.

For harmonic excitation *z_e_ = z*_0_cos (*ωt*), the absolute vibration displacement of the mass *M_i_* is *z_d_*. Then, the kinetic energy of the ML-QZS system is given as

(A1) $$T=\frac{1}{2}M_{i}{\dot{z}}_{d}^{2}$$

and the potential energy *V* is given as

(A2) $$V=\int_{z_{m}}^{z_{r}}\left(F_{R}-M_{i}g\right)dz=\frac{1}{2}k{\left(z_{r}-z_{m}\right)}^{2}+\int_{z_{m}}^{z_{r}}F_{M}dz-M_{i}gz_{r}$$

where *z_r_* is the relative displacement with respect to the base as *z_r_ = z_d_* − *z_e_*, *z_m_* is the initial compression of the vertical spring as *z_m_ = M*_1_ *g*/*k*, *M_i_* is the load mass for the *ith* double-ring layer in the ML-QZS isolator. When *M*_1_ = 0, the initial compression deformation equals zero as *z_m_* = 0 and the initial position is at the equilibrium in the first double-ring layer. Making a general survey of the proposed ML-QZS isolator, there is only a linear bearing assembled in the vibration direction, which provides a damping effect. Assuming the damping coefficient of the linear bearing is *c*, the dissipative force is given as

(A3) $$Q=-\frac{\partial W_{c}}{\partial z_{r}}=-\frac{\partial \left(c{\dot{z}}_{r}z_{r}\right)}{\partial z_{r}}=-c{\dot{z}}_{r}$$

The dynamic equation at the equilibrium in each double magnetic ring layer can be simplified as

(A4) $$M_{i}{\ddot{z}}_{r}^{\left(i\right)}+F_{R}\left(z_{r}^{\left(i\right)}\right)+c{\dot{z}}_{r}^{\left(i\right)}=-M_{i}{\ddot{z}}_{e}$$

where $z_{r}^{\left(i\right)}$ is the relative displacement in the *ith* double-ring layer as $z_{r}^{\left(i\right)}$ *= z_d_* − (*i −* 1)*dis*.

For the displacement excitation from the base as *z_e_ = z*_0_cos (*ωt*), the expression of $z_{r}^{\left(i\right)}$ is assumed to as the harmonic vibration as

(A5) $$z_{r}^{\left(i\right)}=a\sin(\omega t+\theta )$$

According to the Averaging Method, the differential equations of amplitude and phase angle are averaged over the period 0 to 2*π*, written as

(A6) $$\left\{\begin{array}{l} \dot{a}=\frac{1}{2\pi \omega }\int_{0}^{2\pi }\left[F_{\zeta }+F_{isp}\sin\left(\phi -\theta \right)\right]\cos\phi d\phi =\frac{1}{2\pi \omega }\left[Y\left(a,\omega \right)-F_{isp}\sin\theta \right]\\ \dot{\theta }=-\frac{1}{2\pi \omega a}\int_{0}^{2\pi }\left[F_{\zeta }+F_{isp}\sin\left(\phi -\theta \right)\right]\sin\phi d\phi =\frac{-1}{2\pi \omega a}\left[P\left(a,\omega \right)+F_{isp}\cos\theta \right] \end{array}\right.$$

where *θ* = arctan(−*Y* (*a*,*ω*)/*P* (*a*,*ω*)), *ϕ* = *ωt* + *θ*, *F_isp_* = *z*_0_*ω*^2^, *F_ζ_* = *aω*^2^sin*ϕ* – *caω*cos *ϕ*/*M_i_* − *F_R_* (*a*sin *ϕ*)/*Mi*, *Y* (*a*,*ω*) = −*caω*/*M_i_* − $\int_{0}^{2\pi }F_{R}(asin\phi )cos\phi d\phi$, and *P* (*a*,*ω*) = −*aM_i_ω*^2^ − $\int_{0}^{2\pi }F_{R}(asin\phi )sin\phi d\phi$. The steady-state responses, stability, and bifurcation condition are analyzed in Section 3.1.

Then, in order to obtain the critical condition for single- and multi-steady states, the bifurcation analysis is given. For the differential equations of the amplitude and phase, as shown in Equation (A6), we introduce a perturbation of the steady-state solution as

(A7) $$\left\{\begin{array}{l} a=a_{0}+\Delta a\\ \theta =\theta_{0}+\Delta \theta \end{array}\right.$$

where *a*_0_ and *θ*_0_ are solved using Equation (16). Substituting the perturbation solution (A7) into Equation (A6), the following differential equations can be obtained:

(A8) $$\left\{\begin{array}{l} {\dot{a}}_{0}+\Delta \dot{a}=\frac{1}{2\pi \omega }\left[Y\left(a_{0}+\Delta a,\omega \right)-F_{isp}\sin\left(\theta_{0}+\Delta \theta \right)\right]\\ {\dot{\theta }}_{0}+\Delta \dot{\theta }=\frac{-1}{2\pi \omega a}\left[P\left(a_{0}+\Delta a,\omega \right)+F_{isp}\cos\left(\theta_{0}+\Delta \theta \right)\right] \end{array}\right.$$

Expanding the functions *Y*, *P*, sin(*θ*_0_ + Δ*θ*) and sin(*θ*_0_ + Δ*θ*) in Equation (A8) around *a*_0_ and *θ*_0_, since *a*_0_ and *θ*_0_ satisfy Equation (A6), the differential equations for the perturbation states Δ*a* and Δ*θ* are given as

(A9) $$\Delta \dot{a}=J\Delta a$$

where **Δa** = [Δ*a*, Δ*θ*]*^T^* and

(A10) $$J=\left[\begin{array}{cc} a_{11} & a_{12}\\ a_{21} & a_{22} \end{array}\right]$$

With

(A11) $$\begin{array}{l} \;a_{11}=\frac{-c}{2\pi M_{i}\omega },\\ a_{12}=\frac{-F_{isp}\cos\theta_{0}}{2\pi \omega }=\frac{F_{isp}}{2\pi \omega }\frac{P\left(a_{0},\omega \right)}{F_{isp}}=\frac{M_{i}a_{0}\omega^{2}-\int_{0}^{2\pi }F_{R}\left(a_{0}\sin\phi \right)\sin\phi d\phi }{2\pi M_{i}\omega },\\ a_{21}=\frac{-M_{i}a_{0}\omega^{2}+\int_{0}^{2\pi }K_{R}\left(a_{0}\sin\phi \right)\sin\phi d\phi }{2\pi M_{i}a_{0}\omega },\\ a_{22}=\frac{-F_{isp}\sin\theta_{0}}{2\pi a_{0}\omega }=\frac{F_{isp}}{2\pi \omega }\frac{Y\left(a_{0},\omega \right)}{F_{isp}}=\frac{-c}{2\pi M_{i}}. \end{array}$$

## Appendix C

Appendix C shows the comparison of critical excitation amplitude *z*_0_ between the single-ring model and the double-ring QZS model for the occurrence of multi-steady states and the vibration jumping phenomenon.

As shown in Figure A2, all critical excitation amplitudes of the double-ring model for different *dis* are larger than that of the single-ring model, which proves the superiority of the proposed structure. With the increase of *dis*, the critical excitation amplitude increases, as the damping coefficient *c* = 1.0. When *dis* = 40 mm and the damping coefficient is small, the critical excitation amplitude decreases. According to the discussion in Section 2.2, with the increase of *dis*, the stiffness at the equilibrium point becomes excessively high, which causes the softened spring characteristic. Due to the softened spring characteristic, the transmissibility curve has a left deviation phenomenon, which reduces the softened spring characteristic, as shown in Figure A6 (e). Therefore, it is necessary to reasonably select *dis* to prevent left deviation and increase critical excitation replication.

## Appendix D

Appendix D shows the two dynamic indices, displacement transmissibility at 6 Hz, and the vibration transient attenuation time for different values of damping coefficient *c* applied in the isolation mount.

Figure A3 shows the effect of different values *c* on the transient time *t_s_* (blue points) and the transmissibility at *ω_a_* = 6 Hz (red points), respectively.

As shown in Figure A3, for different damping coefficients *c*, the change rules of the two indices *t_s_* and *T_e_* are different. For different values of *c* = 0.3, 0.45, 0.65, 0.9, and 1.2, marked by green points, the comparison of the transient response *t_s_* and transmissibility *T_e_* is given in Figure A4.

Therefore, a larger damping coefficient results in faster vibration transient attentuation, while inducing higher displacement transmissibility *T_e_* in the high-frequency band. An optimization criterion to coordinate the dynamic performances should be established.

## Appendix E

Appendix E shows the two dynamic indices, displacement transmissibility at 6 Hz, and the vibration transient attenuation time for different structural parameter *dis* applied in the isolation mount.

Figure A5 shows the effect of different parameter *dis* on the transient time *t_s_* (blue points) and the transmissibility at *ω_a_* = 6 Hz (red points), respectively.

As shown in Figure A5, for different *dis* values, the patterns of change for the two indices *t_s_* and *T_e_* are different. For different parameter values of *dis* = 30.0 mm, 32.0 mm, 35.0 mm, 37.5 mm, and 40.0 mm, marked by green points, the comparison of the transient response *t_s_* and transmissibility *T_e_* is given in Figure A6.

A larger parameter *dis* results in slower vibration transient and induces lower displacement transmissibility *T_e_* in the high-frequency band when *dis* is less than 32 mm. However, when *dis* is greater than 32 mm, a larger parameter *dis* leads to faster vibration transient ad induces higher displacement transmissibility *T_e_* in the high-frequency band. Therefore, as the parameter *dis* changes, the two dynamic indices are contradictory. An optimization criterion to coordinate the dynamic performances should be established.

## References

[1] Ding J., Chang Y., Chen P., Zhuang H., Ding Y., Lu H., Chen Y. (2020). Dynamic modeling of ultra-precision fly cutting machine tool and the effect of ambient vibration on its tool tip response. Int. J. Extrem. Manuf..

[2] Miao H., Wang C., Li C., Song W., Zhang X., Xu M. (2023). Vibration characteristics and reliability analysis of roller linear guideway workbench. Nonlinear Dyn..

[3] Zhang Q., Shi W., Ouyang Y. (2025). Vibration isolation performance of quasi constant natural frequency isolation pads associated with test verification. Eng. Struct..

[4] Liu X.L., Shangguan W.B., Jing X., Ahmed W. (2016). Vibration isolation analysis of clutches based on trouble shooting of vehicle accelerating noise. J. Sound Vib..

[5] Shi X., Zhou H., Zhou C., Guo Z., Ren Z. (2024). Design and mechanical properties of metal rubber secondary multidirectional vibration isolation system under random vibration. Nonlinear Dyn..

[6] Yu Y., Naganathan N.G., Dukkipati R.V. (2001). A literature review of automotive vehicle engine mounting systems. Mech. Mach. Theory.

[7] Liu C., Jing X., Daley S., Li F. (2015). Recent advances in micro-vibration isolation. Mech. Syst. Signal Process..

[8] Yan B., Ma H., Zhao C., Wu C., Wang K., Wang P. (2018). A vari-stiffness nonlinear isolator with magnetic effects: Theoretical modeling and experimental verification. Int. J. Mech. Sci..

[9] Carrella A., Brennan M., Waters T. (2007). Static analysis of a passive vibration isolator with quasi-zero-stiffness characteristic. J. Sound Vib..

[10] Carrella A., Brennan M., Waters T., Shin K. (2008). On the design of a high-static–low-dynamic stiffness isolator using linear mechanical springs and magnets. J. Sound Vib..

[11] Tang B., Brennan M.J. (2014). On the shock performance of a nonlinear vibration isolator with high-static-low-dynamic-stiffness. Int. J. Mech. Sci..

[12] Hao Z., Cao Q., Wiercigroch M. (2016). Two-sided damping constraint control strategy for high-performance vibration isolation and end-stop impact protection. Nonlinear Dyn..

[13] Li M., Li X., Gan C., Zeng J., Zhao L., Ding H., Wei K., Zou H. (2023). Human motion energy harvesting backpack using quasi-zero stiffness mechanism. Energy Convers. Manag..

[14] Mao X., Yin M., Ding H., Geng X., Shen Y., Chen L. (2022). Modeling, analysis, and simulation of X-shape quasi-zero-stiffness-roller vibration isolators. Appl. Math. Mech..

[15] Li M., Cheng W., Xie R. (2021). A quasi-zero-stiffness vibration isolator using a cam mechanism with user-defined profile. Int. J. Mech. Sci..

[16] Zuo S., Wang D., Zhang Y., Luo Q. (2024). An innovative design of parabolic cam-roller quasi-zero-stiffness isolators for ultralow frequency vibration isolation. Nonlinear Dyn..

[17] Wang Q., Zhou J., Xu D., Ouyang H. (2020). Design and experimental investigation of ultra-low frequency vibration isolation during neonatal transport. Mech. Syst. Signal Process..

[18] Guo M., Tang L., Wang H., Liu H., Gao S. (2023). A comparative study on transient vibration suppression of magnetic nonlinear vibration absorbers with different arrangements. Nonlinear Dyn..

[19] Wang K., Zhou J., Ouyang H., Cheng L., Xu D. (2020). A semi-active metamaterial beam with electromagnetic quasi-zero-stiffness resonators for ultralow-frequency band gap tuning. Int. J. Mech. Sci..

[20] Hou S., Wei J. (2024). A quasi-zero stiffness mechanism with monolithic flexible beams for low-frequency vibration isolation. Mech. Syst. Signal Process..

[21] Liu J., Wang Y., Yang S., Sun T., Yang M., Niu W. (2024). Customized quasi-zero-stiffness metamaterials for ultra-low frequency broadband vibration isolation. Int. J. Mech. Sci..

[22] Lei X., Sun X., Cheng L., Yu X. (2024). A 3D-printed quasi-zero-stiffness isolator for low-frequency vibration isolation: Modelling and experiments. J. Sound Vib..

[23] Gatti G., Ledezma-Ramirez D.F., Brennan M.J. (2023). Performance of a shock isolator inspired by skeletal muscles. Int. J. Mech. Sci..

[24] Li H., Wu Z., Jiang T. (2019). A quasi-zero stiffness vibration isolator based on hybrid bistable composite laminate. Chin. J. Theor. Appl. Mech..

[25] Zeng R., Wen G., Zhou J., Zhao G. (2021). Limb-inspired bionic quasi-zero stiffness vibration isolator. Acta Mech. Sin..

[26] Bian J., Jing X. (2020). Analysis and design of a novel and compact X-structured vibration isolation mount (X-Mount) with wider quasi-zero-stiffness range. Nonlinear Dyn..

[27] Tian R., Wang M., Zhang Y., Jing X., Zhang X. (2024). A concave X-shaped structure supported by variable pitch springs for low-frequency vibration isolation. Mech. Syst. Signal Process..

[28] Yu C., Jiang Q., Fu Q., Yu K., Zhang J., Zhang N. (2023). The X-shaped structure with nonlinear positive stiffness compensation for low-frequency vibration isolation. Int. J. Mech. Sci..

[29] An J., Chen G., Deng X., Xi C., Wang T., He H. (2022). Analytical study of a pneumatic quasi-zero-stiffness isolator with mistuned mass. Nonlinear Dyn..

[30] Ye K., Ji J. (2022). An origami inspired quasi-zero stiffness vibration isolator using a novel truss-spring based stack Miura-ori structure. Mech. Syst. Signal Process..

[31] Liu T., Li A., Zhang H. (2024). Quasi-zero stiffness interval optimization design and dynamics analysis of a new bi-directional horizontal isolation system. Mech. Syst. Signal Process..

[32] Liu X.C., Ding H., Geng X.F., Wei K.X., Lai S.K., Chen L.Q. (2024). A magnetic nonlinear energy sink with quasi-zero stiffness characteristics. Nonlinear Dyn..

[33] Xu J., Yang X., Li W., Zheng J., Wang Y., Fan M., Zhou W., Lu Y. (2020). Design of quasi-zero stiffness joint actuator and research on vibration isolation performance. J. Sound Vib..

[34] Sun X., Wang F., Xu J. (2019). Analysis, design and experiment of continuous isolation structure with Local Quasi-Zero-Stiffness property by magnetic interaction. Int. J. Non-Linear Mech..

[35] Liu C., Zhao R., Yu K., Liao B. (2021). In-plane quasi-zero-stiffness vibration isolator using magnetic interaction and cables: Theoretical and experimental study. Appl. Math. Model..

[36] Kovacic I., Brennan M.J., Waters T.P. (2008). A study of a nonlinear vibration isolator with a quasi-zero stiffness characteristic. J. Sound Vib..

[37] Zuo S., Wang D., Zhang Y., Luo Q. (2022). Design and testing of a parabolic cam-roller quasi-zero-stiffness vibration isolator. Int. J. Mech. Sci..

[38] Sun Y., Zhou J., Thompson D., Yuan T., Gong D., You T. (2020). Design, analysis and experimental validation of high static and low dynamic stiffness mounts based on target force curves. Int. J. Non-Linear Mech..

[39] Zhang Y., Wen H., Hu H., Jin D. (2025). A novel quasi-zero stiffness isolator with designable stiffness using cam-roller-spring-rod mechanism. Acta Mech. Sin..

[40] Yu K., Chen Y., Yu C., Zhang J., Lu X. (2024). A compact nonlinear stiffness-modulated structure for low-frequency vibration isolation under heavy loads. Nonlinear Dyn..

[41] Yan G., Wu Z.Y., Wei X.S., Wang S., Zou H.X., Zhao L.C., Qi W.H., Zhang W.M. (2022). Nonlinear compensation method for quasi-zero stiffness vibration isolation. J. Sound Vib..

[42] Yan G., Qi W.H., Lu J.J., Liu F.R., Yan H., Zhao L.C., Wu Z.Y., Zhang W.M. (2024). Bio-inspired multi-joint-collaborative vibration isolation. J. Sound Vib..

[43] Zhao F., Ji J., Ye K., Luo Q. (2021). An innovative quasi-zero stiffness isolator with three pairs of oblique springs. Int. J. Mech. Sci..

[44] Pu H., Liu J., Wang M., Ding J., Sun Y., Peng Y., Luo J. (2024). Bio-inspired quasi-zero stiffness vibration isolator with quasilinear negative stiffness in full stroke. J. Sound Vib..

[45] Gatti G. (2020). Statics and dynamics of a nonlinear oscillator with quasi-zero stiffness behaviour for large deflections. Commun. Nonlinear Sci. Numer. Simul..

[46] Zhao F., Ji J., Luo Q., Cao S., Chen L., Du W. (2021). An improved quasi-zero stiffness isolator with two pairs of oblique springs to increase isolation frequency band. Nonlinear Dyn..

[47] Zhang Y., Zhu G., Cao Q. (2024). Isolation performances and optimization of triple quasi-zero stiffness isolators. Sci. China Phys. Mech. Astron..

[48] Zhao F., Ji J., Cao S., Ye K., Luo Q. (2024). QZS isolators with multi-pairs of oblique bars for isolating ultralow frequency vibrations. Nonlinear Dyn..

[49] Chen T., Zheng Y., Song L., Gao X., Wang G. (2023). Study on a quasi-zero-stiffness isolator for variable mass load. Appl. Math. Model..

[50] Xu D., Yu Q., Zhou J., Bishop S. (2013). Theoretical and experimental analyses of a nonlinear magnetic vibration isolator with quasi-zero-stiffness characteristic. J. Sound Vib..

[51] Chen R., Li X., Tian J., Yang Z., Xu J. (2022). On the displacement transferability of variable stiffness multi-directional low frequency vibration isolation joint. Appl. Math. Model..

[52] Jiao G., Zeng J., Wang S. (2024). A compact magnetic-curved-spring QZS isolator for supporting uncertain loads. Nonlinear Dyn..

[53] Qi W.H., Yan G., Lu J.J., Liu F.R., Zhao T.Y., Yan H., Zhang W.M. (2024). Local gravity control method for solving load-mismatch issue in isolators. Int. J. Mech. Sci..

[54] Lu J.J., Yan G., Qi W.H., Yan H., Liu F.R., Zhao T.Y., Zhang W.M. (2024). Integrated vibration isolation and actuation via dual nonlinear stiffness regulation. Int. J. Mech. Sci..

[55] Ye K., Ji J., Brown T. (2020). Design of a quasi-zero stiffness isolation system for supporting different loads. J. Sound Vib..

[56] Zhou J., Zhou J., Pan H., Wang K., Cai C., Wen G. (2024). Multi-layer quasi-zero-stiffness meta-structure for high-efficiency vibration isolation at low frequency. Appl. Math. Mech..

[57] Zeng C., Liu L., Hu Y., Zhao W., Xin X., Liu Y., Leng J. (2024). Stair-Stepping Mechanical Metamaterials with Programmable Load Plateaus. Adv. Funct. Mater..

